# Interleukin-10 Is a Promising Marker for Immune-Related Adverse Events in Patients With Non-Small Cell Lung Cancer Receiving Immunotherapy

**DOI:** 10.3389/fimmu.2022.840313

**Published:** 2022-02-09

**Authors:** Haowei Wang, Fei Zhou, Chao Zhao, Lei Cheng, Caicun Zhou, Meng Qiao, Xuefei Li, Xiaoxia Chen

**Affiliations:** ^1^ Department of Medical Oncology, Shanghai Pulmonary Hospital, School of Medicine, Tongji University, Shanghai, China; ^2^ Department of Lung Cancer and Immunology, Shanghai Pulmonary Hospital, School of Medicine, Tongji University, Shanghai, China

**Keywords:** immunotherapy, toxicity, interleukin−10, immune−related adverse events, NSCLC

## Abstract

**Background:**

Immune checkpoint inhibitors (ICIs) brought about a major paradigm shift in non-small cell lung cancer (NSCLC) treatment. However, the use of ICIs is related to an unforeseeable pattern of immune-related adverse events (irAEs). Hence, more precise biomarkers are needed to predict the incidence of irAEs to prevent overtreatment of ICIs and decrease occurrences of irAEs. This study was designed to identify capable clinical features and plasma inflammatory factors for predicting irAEs.

**Methods:**

A total of 67 patients who received ICI monotherapy or ICI-based combination therapy were retrospectively identified. Clinical characteristics and plasma inflammatory cytokines were collected and analyzed to screen potential biological markers associated with irAEs. The chi-square test, Fisher’s test, and the Mann–Whitney U test were performed for the primary analysis. The optimal cutoff value was determined by a receiver operating characteristic (ROC) curve. Univariate and multivariate logistic regression models were used to identify risk factors of irAEs. Univariate and multivariate Cox proportional hazards were also performed.

**Results:**

Out of 67 patients, 40 (59.7%) experienced irAEs, and 7 (10.4%) experienced severe adverse events (grade ≥ 3). Among these analyzed immune profile biomarkers, only interleukin−10 (IL-10) was related to the risk of irAEs. A high baseline IL−10 plasma level (odds ratio (OR) = 5.318, 95% CI 1.174–24.081, p = 0.030) was found to be a tremendous and independent risk factor for the development of irAEs. Also, for the dynamic analysis, upregulation of IL-10 after one cycle of ICI treatment was positively related to the occurrence of irAEs (OR = 5.712, 95% CI 1.088–29.993, p = 0.039). When pneumonitis, the most common irAEs, was analyzed, only baseline high-expression IL-10 was accompanied with the incidence of pneumonitis (OR = 9.969, 95% CI 1.144–86.843, p = 0.037).

**Conclusion:**

Baseline and dynamic IL-10 plasma levels are tremendously and independently related to higher risk in the development of irAEs and could be utilized for medical practice to monitor adverse events in patients with ICI treatment.

## Introduction

During the past years, immunotherapy, targeting immune checkpoint molecules, has led to dramatic advances in oncotherapy, especially in non-small cell lung cancer (NSCLC) treatment (1). However, immune checkpoint inhibitors (ICIs) not only can provide outstandingly durable responses in NSCLC but also is associated with a wide range of immune-related adverse events (irAEs) (2). Although it is still conflicting whether irAEs are accompanied with better treatment outcome (3), these irAEs could be disastrous and extend exposure to immunosuppressive therapies to counteract excessive immune upside risk by ICI therapies (4). Previous studies have reported that irAEs were observed in 51% of patients treated with ICIs (5) and 49% in 623 patients with advanced-stage NSCLC included worldwide (6). Also, among these irAEs, immune-related pneumonitis has been receiving extensive attention owing to its high discontinuation and fatality rate, especially in NSCLC (7). Therefore, the identification of biomarkers to predict irAEs and pneumonitis occurrence owing to ICIs is essential to increase the benefits during ICI treatment.

By eliminating the restraint of T-cell function, ICIs promote T cell-mediated effects on cancers. Nonetheless, they could also enhance the activity of mediators and cell sets that function against host tissues and potentially promote autoimmune activity (3). In addition, cytokines may take a role in the pathophysiology of irAEs. Previous studies determined that the level of interleukin (IL)-17 increased in patients with immunotherapy-associated colitis (8) and that increased levels of IL-17 have been detected in preclinical models of colitis (9). A prospective study reported that baseline IL-6 serum levels were independently correlated with a higher risk of severe toxicity, which thus could be used in the clinic to conduct personalized toxicity monitoring for melanoma patients receiving immunotherapy (10).

The purpose of this present study was to identify easily accessible biomarkers to predict the occurrence of irAEs. Given that these biomarkers in the peripheral blood are easily available and can be highly standardized and undergo repeated evaluations, it is particularly ideal to evaluate these biomarkers. With this project, our team members collected and analyzed the clinical characteristics and peripheral blood biomarkers including blood cells and a set of functionally selected cytokines both at baseline (before ICI treatment) and during dynamic monitoring (with ICI treatment).

## Methods

### Patients and Therapy

In Shanghai Pulmonary Hospital, data of patients with advanced-stage NSCLC with ICI therapy between August 20, 2018, and May 12, 2020, were recorded. The eighth edition of the TNM staging system was utilized to stage these included patients (11). All patients received ICIs as monotherapy or combination therapy, despite treatment lines.

### Data Collection

Electronic medical characteristics were collected, including gender, age, smoking history, pathology, stage, Eastern Cooperative Oncology Group Performance Status (ECOG PS), PD-L1, ICI treatment strategy, and immunotherapy treatment line. Brain, liver, and bone metastases were also included. In addition, to explore the significance of the peripheral blood biomarkers, leukocyte, neutrophil, and lymphocyte were also recorded. IrAEs were defined depending on the fifth classification of the Common Terminology Criteria for Adverse Events (CTCAE). All irAEs included were confirmed by two researchers. Patients were followed up until January 27, 2021.

### Cytokine Measurements

Plasma was extracted from the peripheral blood and frozen at −80°C immediately after extraction. Cytokines (IL-1β, IL-2, IL-4, IL-6, IL-8, IL-10, IL-12p70, IL-13, IFN γ, and tumor necrosis factor (TNF)-α) were quantified using the human V-Plex Pro-inflammatory Panel 1 Kit (Meso Scale Discovery, Forestville, CA, USA). Since IL-1β and IL-13 fell below the detection threshold after treatment, we analyzed the relationship between the dynamic expression levels of eight cytokines during immunotherapy.

### Statistical Analysis

The categorical variables were analyzed with the chi-square test, Fisher’s test, and the Mann–Whitney U test performed for continuous variables. A receiver operating characteristic (ROC) curve was performed to explore the analysis and determine the best cutoff value for irAEs and pneumonitis. Logistic regression models were used for identifying risk factors of irAEs. Univariate and multivariate Cox proportional hazards were also performed. For RNA-seq data of lung cancer from The Cancer Genome Atlas (TCGA), the count value of gene expression was downloaded using R package GDCRNATools (12) with 1,014 tumor samples and 108 normal samples obtained in this study. The single-sample gene set enrichment analysis (ssGSEA) (13) was performed to quantify each immune-related cell infiltration (14). SPSS software (V 26.0) and RStudio software (V4.0.1) were used for statistical analysis.

## Results

### Patient Characteristics and Immune-Related Adverse Event Profile

**Figure 1:**
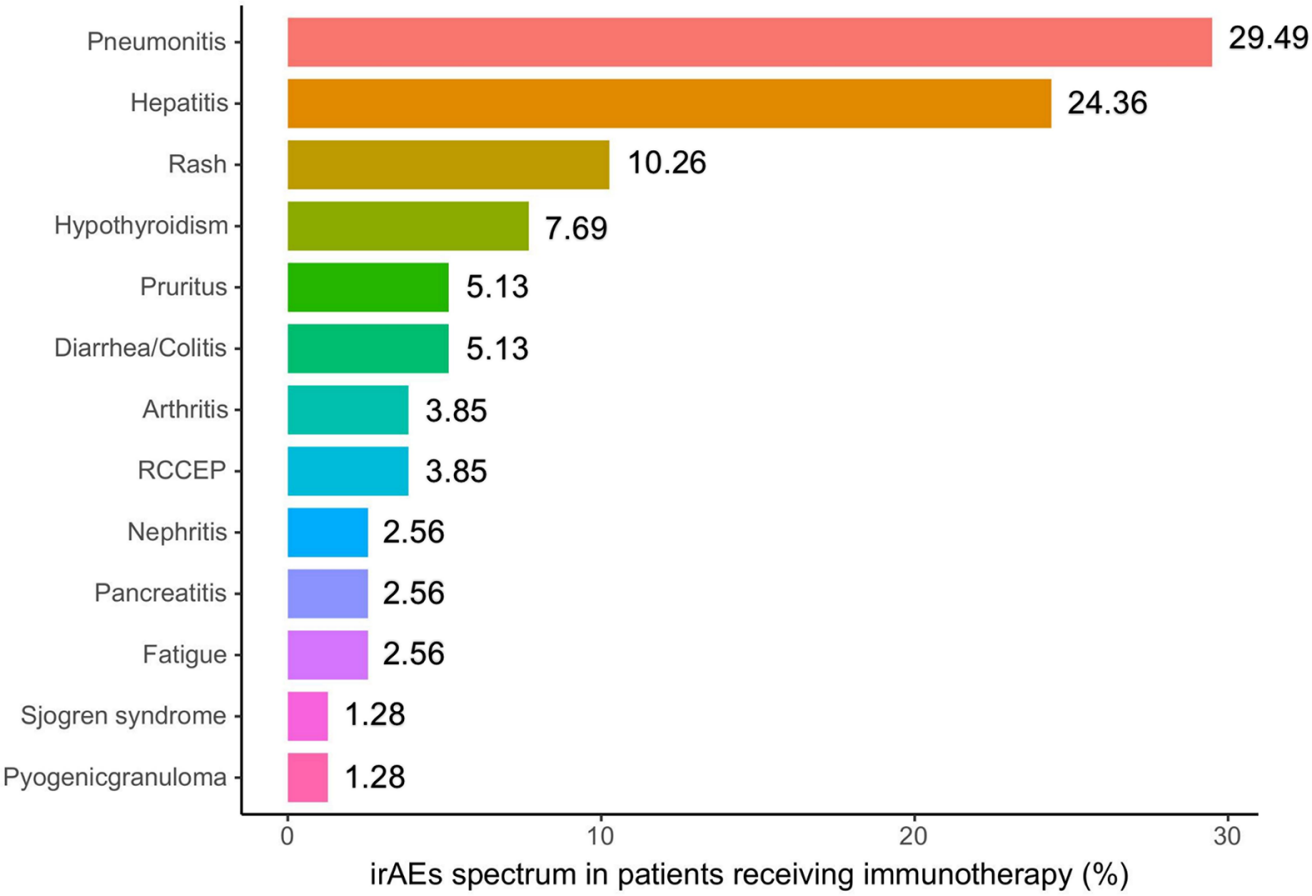
IrAE spectrum in patients receiving immunotherapy. Rates of individual diseases in all included irAEs (n = 78). irAEs, immune-related adverse events; RCCEP, reactive cutaneous capillary endothelial proliferation.

The clinical characteristics of the 67 patients and baseline blood biomarker levels were incorporated in this study and summarized in **Table 1**. The median age at baseline was 65 (40 to 89) years, 13.4% of these patients were female, and 44% were never-smokers. Among them, 50.7% of patients were diagnosed with lung adenocarcinoma, and others were squamous. PD-L1 was detected in 57.5% of these patients. All patients were treated with immunotherapy including monotherapy (56.7%) and combination therapy (43.3%). Of the patients, 27% received ICI treatment as a first-line strategy. No patients had previously received ICI treatments. When regard to metastatic sites, baseline incidence of brain metastasis was 10.4%, liver metastasis was 6%, and bone metastasis was 25%. Also, the median follow-up time was 6.1 months. The irAE profile is shown in **Figure 1**. Of 67 patients, 40 patients (59.7%) developed one or more events for a total of 78 irAEs. The most common irAEs were pneumonitis (29.49%) and hepatitis (24.36%). Of note, 7 patients (10.45%) experienced a severe adverse event (grade ≥ 3). When considering blood biomarkers, the levels of leukocyte, neutrophil, and lymphocyte did not have statistical significance in patients with irAEs or not (**Table 1**), and only IL-10 levels showed evident correlation with irAEs (p = 0.016) among plasma inflammatory factors (**Table 1**).

**Table 1:** Baseline clinical characteristics of patients who received ICIs.

Characteristics	All (%)	Without irAEs (%)	With irAEs (%)	p
Gender				0.410
Female	9 (13.4)	2 (7.4)	7 (17.5)	
Male	58 (86.6)	25 (92.6)	33 (82.5)	
Age (years)				0.582
<65	35 (52.2)	13 (48.1)	22 (55)	
≥65	32 (47.8)	14 (51.9)	18 (45)	
Smoking history				0.025
Never	44 (65.7)	22 (81.5)	22 (55)	
Anytime	23 (34.3)	5 (18.5)	18 (45)	
Pathology				0.032
Squamous	34 (50.7)	18 (66.7)	16 (40)	
Adenocarcinoma	33 (49.3)	9 (33.3)	24 (60)	
Stage				0.365
Postoperative recurrence	16 (23.9)	8 (29.6)	8 (20)	
III/IV	51 (76.1)	19 (70.4)	32 (80)	
ECOG PS				0.654
0	2 (3)	0 (0)	2 (5)	
≥1	65 (97)	27 (100)	38 (95)	
PD-L1				0.385
Not detected	17 (42.5)	9 (50)	8 (36.4)	
Detected	23 (57.5)	9 (50)	14 (63.6)	
treatment lines				0.952
1	27 (40.3)	11 (40.7)	16 (40)	
≥1	40 (59.7)	16 (59.3)	24 (60)	
Treatment strategy				0.509
Monotherapy	38 (56.7)	14 (51.9)	24 (60)	
Combination therapy	29 (43.3)	13 (48.1)	16 (40)	
Brain metastasis				0.794
No	60 (89.6)	25 (92.6)	35 (87.5)	
Yes	7 (10.4)	2 (7.4)	5 (12.5)	
Liver metastasis				0.242
No	63 (94)	27 (100)	36 (90)	
Yes	4 (6)	0 (0)	4 (10)	
Bone metastasis				0.969
No	42 (62.7)	17 (63)	25 (62.5)	
Yes	25 (37.3)	10 (37)	15 (37.5)	
Blood biomarkers, median (1st quartile, 3rd quartile)				
Leukocyte	6.57 (5.71, 8.46)	6.39 (4.90, 8.67)	6.57 (5.78, 8.17)	0.520
Neutrophil	4.48 (3.47, 6.05)	4.56 (3.09, 6.28)	4.48 (3.75, 5.35)	0.763
Lymphocyte	1.36 (1.11, 1.94)	1.62 (1.13, 2.01)	1.24 (1.10, 1.84)	0.154
IFN-γ	9.15 (6.05, 15.86)	8.70 (4.87, 15.86)	9.23 (6.71, 16.89)	0.733
IL-10	1.15 (0.72, 1.92)	0.92 (0.57, 1.61)	1.51 (0.86, 2.28)	0.016
IL-12	0.16 (0.08, 0.27)	0.11 (0.09, 0.18)	0.19 (0.08, 0.37)	0.098
IL-13	1.44 (1.04, 2.15)	1.38 (0.89, 1.80)	1.51 (1.06, 2.60)	0.238
IL-1	0.13 (0.05, 0.29)	0.13 (0.06, 0.26)	0.12 (0.05, 0.33)	0.872
IL-2	0.26 (0.16, 0.40)	0.23 (0.10, 0.39)	0.26 (0.17, 0.46)	0.124
IL-4	0.02 (0.01, 0.03)	0.02 (0.01, 0.03)	0.02 (0.01, 0.03)	0.823
IL-6	3.66 (1.81, 6.73)	3.38 (1.68, 6.38)	3.72 (1.81, 6.90)	0.759
IL-8	5.47 (3.48, 8.86)	5.69 (3.48, 8.28)	5.13 (3.09, 10.73)	0.858
TNF-α	4.93 (3.81, 6.19)	5.02 (3.40, 6.94)	4.89 (3.97, 6.13)	0.808

ICIs, immune checkpoint inhibitors; irAEs, immune-related adverse events; ECOG PS, Eastern Cooperative Oncology Group Performance Status.

### Association Between Baseline IL-10 and Immune-Related Adverse Events

**Figure 2:**
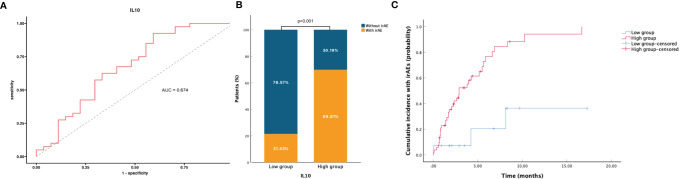
Association between baseline blood IL-10 and irAEs. **(A)** ROC curve analysis of IL-10 and irAEs, AUC = 0.674. **(B)** The probability of occurrence of IrAEs in patients with low IL-10 and high IL-10. **(C)** Accumulative incidence of irAEs with patients during ICI therapy. Patients who died or have missing follow-ups were defined as censored values. ROC, receiver operating characteristic; AUC, area under the curve; irAEs, immune-related adverse events; ICI, immune checkpoint inhibitor.

**Figure 3:**
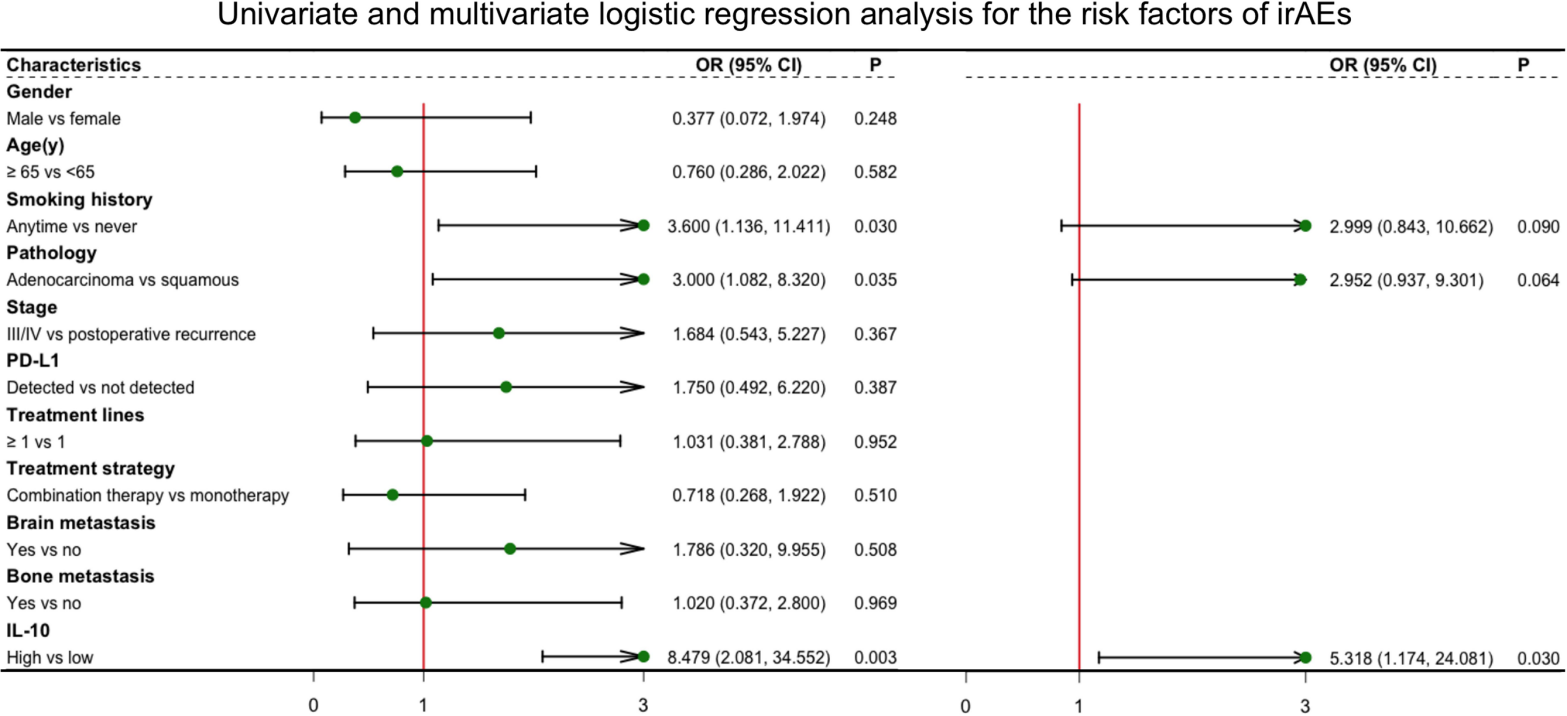
Logistic regression with univariate and multivariate analyses for the risk factors of irAEs. Characteristics in univariate models with p-value <0.05 were included in multivariate analysis. irAEs, immune-related adverse events.

**Figure 4:**
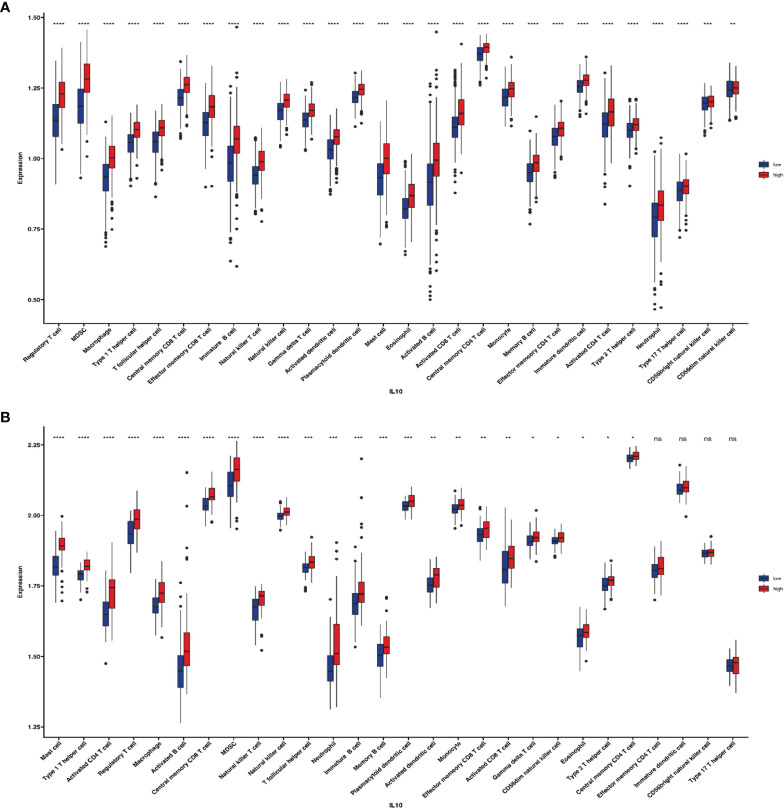
The role of IL-10 in the immune-related cell infiltration. **(A)** Twenty-eight immune-related infiltration cells between high and low IL-10 groups in non-small cell lung tumor samples. **(B)** Twenty-eight immune-related infiltration cells between high and low IL-10 groups in normal samples (ns: p ≥ 0.05, *p < 0.05; **p < 0.01; ***p < 0.001; ****p < 0.0001).

All blood biomarkers were evaluated in the ROC curve analysis (**Supplementary Figure 1**) and the Mann–Whitney U test (**Table 1**) for the predictive of irAEs, and only IL-10 was found to have significance (area under the curve (AUC) = 0.674, p = 0.016) (**Figure 2A**). Based on these results, we chose IL-10 for further analysis. The optimal cutoff value of the baseline IL-10 to differentiate the occurrence of irAEs was 0.704 pg/ml (sensitivity = 92.5%, specificity = 40.7%). Therefore, patients with baseline IL-10 ≥0.704 were defined as the high IL-10 group, with others defined as the low IL-10 group. Univariate and multivariate logistic regression analyses were performed to reveal that IL-10 (odds ratio (OR) = 5.318, 95% CI 1.174–24.081, p = 0.030) (**Figure 3**) was significantly and independently associated with the occurrence of irAEs. Also, the incidence of irAEs was higher in the high IL-10 group compared with the low IL-10 group (69.81% vs. 21.43%, p = 0.001) (**Figure 2B**). There is little evident difference (p = 0.780) in the distribution of severity among the high IL-10 and low IL-10 groups (**Supplementary Figure 3A**). The median time of irAEs was 2.13 months (range 0.03–16.7 months). The median time was 2.03 months in the high IL-10 group (range 0.37–16.7 months) and 4.23 months in the low IL-10 group (range 0.03–8.13 months). Furthermore, the accumulative incidence of irAEs (**Figure 2C**) showed a significant difference in different groups (p = 0.003). Taking the occurrence time of irAEs into consideration, univariate and multivariate Cox proportional hazards regression analyses (**Supplementary Table 1**) also indicated that only IL-10 was associated with the incidence of irAEs (hazard ratio (HR) = 4.458, 95% CI 1.329–14.953, p = 0.015). In order to explore the difference of identified immune cell infiltration, we evaluated the landscape of 28 immune-related cells in both tumor (n = 1014) and normal (n = 108) samples. We used the value of the median of IL-10 to define the high IL-10 group and low IL-10 group. As shown in **Figure 4**, we found that all immune cells presented significant difference in infiltration between the high IL-10 group and low IL-10 group in tumor samples, and most of these cells were also significantly enriched in high IL-10 with normal tissues. This indicated that a high level of IL-10 may be correlated with an activated inflammatory environment.

### Association Between Dynamic IL-10 and Immune-Related Adverse Events

The plasma of 36 patients was collected both at baseline and during treatment and was used to analyze the relationship between the dynamic change of cytokines and the incidence of irAEs. The median change was used to differentiate between the high and low groups for these cytokines, the chi-square test was performed (**Supplementary Table 2**), and IL-10 was found to differ significantly (p = 0.015). Furthermore, univariate and multivariate analyses (**Supplementary Table 3**) determined that high IL-10 during treatment was still associated with the occurrence of irAEs (OR = 5.712, 95% CI 1.088–29.993, p = 0.039).

### Association Between Baseline IL-10 and Immune Checkpoint Inhibitor-Related Pneumonitis

**Figure 5:**
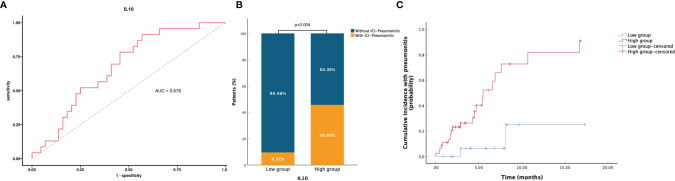
Association between baseline blood IL-10 and ICI pneumonitis. **(A)** ROC curve analysis of IL-10 and ICI pneumonitis, AUC = 0.678. **(B)** The probability of ICI pneumonitis in patients with high IL-10 and low IL-10. **(C)** Accumulative incidence of ICI pneumonitis among patients during ICI therapy. Patients who were lost to follow-up or died were defined as censored value. ICI-pneumonitis, immune checkpoint inhibitor-related pneumonitis; ROC, receiver operating characteristic; AUC, area under the curve.

**Figure 6:**
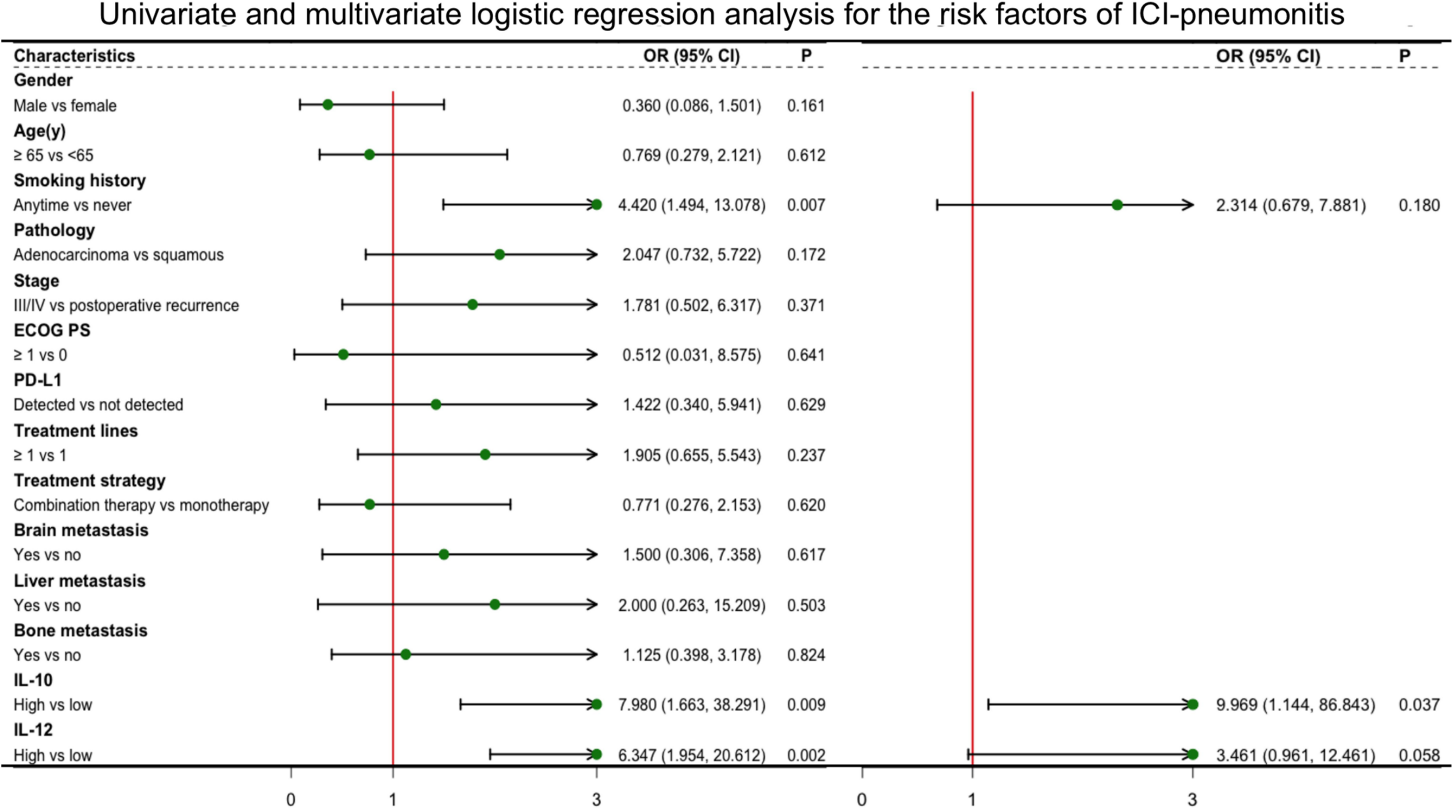
Univariate and multivariate logistic regression analyses for the risk factors of ICI-pneumonitis. Characteristics in univariate models with p-value <0.05 were included in multivariate analysis. ICI-pneumonitis, immune checkpoint inhibitor-related pneumonitis.

Considering that pneumonia was the most common irAE in this study and one of the most worrying adverse reactions in advanced NSCLC patients during ICI therapy (7). Therefore, in addition to identifying IL-10 as a reliable predictor for the irAEs, we hope to find out if IL-10 could also play an eligible role in predicting pneumonia during ICI therapy. The baseline clinical characteristics of patients in pneumonitis are stated in **Supplementary Table 4**. Smoking history (p = 0.006), IL-10 (p = 0.017), and IL-12 (p = 0.009) were found have significance in distinguishing the occurrence of pneumonitis. Both IL-10 (**Figure 5A**) and IL-12 (**Supplementary Figure 2**) were used in the ROC to determine the most appropriate cutoff value of the high and low groups. Univariate and multivariate logistic regression analyses were utilized (**Figure 6**), and IL-10 (OR = 9.969, 95% CI 1.144-86.843, p = 0.037) was credible and independently related to the occurrence of pneumonitis. Besides, the incidence of pneumonia was higher in the high baseline IL-10 group when compared with the low group (45.65% vs. 9.52%, p = 0.004) (**Figure 5B**). There was no evident difference in high and low IL-10 in severity grade (p = 0.786) (**Supplementary Figure 3B**). Also, Cox proportional hazards regression model (**Supplementary Table 5**) further verified the value of IL-10 (HR = 14.015, 95% CI 1.794–109.507, p = 0.012). The median time in the high IL-10 group was 4.5 months (range 0.70–10.73 months) and in the low IL-10 group was 4.66 months (range 1.70–7.63 months). Moreover, the cumulative incidence of pneumonitis (**Figure 5C**) showed a significant difference in different groups (p = 0.002). For the reason of few occurrences of pneumonitis in 37 patients during dynamic monitoring, we did not find any difference in the dynamic changes of these cytokines in patients with pneumonia or not (**Supplementary Tables 6**, **7**).

## Discussion

Impressive agent activity of various ICIs has led to regulatory approvals for multiple agents in various solid tumor indications (15–19). IrAEs from ICI differ from cytotoxicity or toxicity caused by molecular targeting agents. The time of toxicity may be delayed rather than following periodic patterns such as traditional cytotoxicity. Mechanisms have yet to be determined, and even with the same agent, it is likely to be heterogeneous between patients.

These irAEs are widely distributed in terms of affected organs and severity. Dermatology, endocrinology, neurology, gastrointestinal, respiratory, and musculoskeletal toxicity may occur individually or in constellations. More than two-thirds of cancer immunotherapy-related irAEs cases are ICI related, with three drugs (ipilimumab, nivolumab, and pembrolizumab) accounting for nearly 60% of reported cases (20). A preceding clinical trial reported that patients who were treated with nivolumab alone, ipilimumab alone, or nivolumab plus ipilimumab developed grade 3 or 4 irAEs with a rate of 21%, 28%, and 59%, respectively. Furthermore, four patients passed away owing to severe irAEs (21). Most are self-limiting or addressing immunosuppressants such as corticosteroids. ICI treatment can regularly continue through close supervision to avoid more serious irAEs. However, severe irAEs could be related to a grave decline in function of these organs and quality in daily life, and especially fatal results have been reported.

If these irAEs were early detected and properly dealt with, most of them are moderate and reversible. Therefore, it is essential to look for biomarkers in predicting the incidence of irAEs. Compared with many biomarkers of tumor response, there were fewer investigations of biomarkers of tumor irAEs (22). Previous studies have reported that body composition parameters, such as sex, IL-6, IL-17, CXCL5, blood cell counts, autoantibodies, T-cell repertoire, and gut microbiome were relevant to the occurrence of irAEs. Cytokines were reported as the important effect and messenger molecules in the human immune system (23). They are deeply involved in the immune response to infection and inflammation, preventing or contributing to diseases such as allergies, autoimmunity, and tumors. However, with regard to these cytokines, different cytokines and even the same cytokine may play different roles in predicting the irAEs in different studies (10, 24).

In order to explore the correlation of these cytokines with irAEs, a set of functionally selected cytokines was detected and analyzed. Among these considered biomarkers, baseline and dynamic levels of IL-10 are known to have a crucial role in anti-inflammatory and immunosuppressive effects and can stratify the risk of irAEs, particularly, higher levels of IL-10, indicating the higher risk of irAEs.

IL-10 is reported as an immunomodulatory cytokine and could be provided by different cell types in humans, such as CD4^+^ T cells, CD8^+^ T cells, B cells, and monocytes (25, 26). Recently, macrophages were expanded to IL-10-producing cells (27, 28) and some non-hematopoietic cells, including epithelial (29) and tumor cells (30). In addition to being provided by the above cells, IL-10 functionally targets diverse cells, resulting in even different paradoxical roles in functions with immunity and cancer. Some studies found that IL-10 induced T cell anergy, while others confirmed that IL-10 could stimulate the proliferation of mature CD8+ T cells and can increase the cytolytic activity of the T cells. The faint effects of IL-10 were reported in NK cells as well. NK cell expression was suppressed by IL-10, although IL-10 can also increase NK cytolytic activity. However, IL-10 promotes the survival of B cells and proliferation as well as differentiation, which have been much clear. In addition, IL-10 has been well-documented to restrain MHC class II expression. In our study, we found that a high level of IL-10 was correlated with activated inflammatory environment in both tumor and normal samples.

To our knowledge, this is the first study to investigate the predictive value of baseline and dynamic plasma IL-10 for irAEs in NSCLC patients. Our results suggested that patients with a high level of baseline IL-10 have a higher risk of irAEs (OR = 5.318, p = 0.030). Also, during ICI treatment, the dynamic change of IL-10 was still associated with the occurrence of irAEs. With regard to pneumonitis as the most frequent event among these irAEs, the baseline level of IL-10 plays a reliable role (OR = 9.969, p = 0.037) in predicting the incidence.

However, there are still some limitations. First, this was a single-center retrospective evaluation, and 67 patients were included in our analysis, suggesting that there may have been information bias that needs to be verified in more large and multicenter cohorts. Second, the exact aspects of irAEs were related to ICI drugs, and the predictive role of IL-10 may need to be verified in these ICI drugs to help clinicians personalize immune-toxicity surveillance. Third, we did not explore what the specific mechanisms IL-10 plays in these patients with irAEs because IL-10 may not only serve as a reliable predictor of irAEs but also serve as target therapy to eliminate or reduce these irAEs.

## Conclusion

This study analyzed the correlation of a set of functionally selected blood cytokines and the incidence of irAEs. The multivariate analysis indicated that baseline and dynamic blood IL-10 is a promising biomarker for irAEs. These findings suggested that patients with a high level of IL-10 should be carefully monitored for toxicity during ICI therapy. Further prospective studies in larger cohorts are necessary to validate our results.

## Data Availability Statement

The original contributions presented in the study are included in the article/**Supplementary Material**. Further inquiries can be directed to the corresponding author.

## Ethics Statement

The studies involving human participants were reviewed and approved by The Ethics Committee of Shanghai Pulmonary Hospital, Tongji University. The patients/participants provided their written informed consent to participate in this study.

## Author Contributions

HW and XC designed this study and drafted the manuscript. MQ and XL collected the data. FZ, CZ, and LC analyzed the data. CCZ gave critical comments. XC and HW revised the paper. All authors contributed to this article and approved the submitted version.

## Funding

This study was partly supported by grants from the Shanghai Science and Technology Innovation Action Plan Medical Innovation Research Project (No. 21Y11913600), Shanghai Nature Foundation Project (No. 21ZR1453200), Clinical Research Project of Shanghai Pulmonary Hospital (No. Fk18002), Shanghai Innovative Collaboration Project (No. 2020CXJQ02), National Natural Science Foundation of China (No. 81972169), and Establishment and promotion of multidisciplinary collaborative diagnosis and treatment system for non-infectious diseases in the lungs.

## Conflict of Interest

The authors declare that the research was conducted in the absence of any commercial or financial relationships that could be construed as a potential conflict of interest.

## Publisher’s Note

All claims expressed in this article are solely those of the authors and do not necessarily represent those of their affiliated organizations, or those of the publisher, the editors and the reviewers. Any product that may be evaluated in this article, or claim that may be made by its manufacturer, is not guaranteed or endorsed by the publisher.

## Supplementary Material

Supplementary Figure 1 ROC curve analysis of all blood biomarkers included and irAEs.

Supplementary Figure 2 ROC curve analysis of all blood biomarkers included and ICI-pneumonitis.

Supplementary Figure 3 Grade distribution of irAEs **(A)** and ICI-pneumonitis **(B)** in patients with high IL-10 and low IL-10 group.
