# Supplementary figures and images for: Interleukin-10 Is a Promising Marker for Immune-Related Adverse Events in Patients With Non-Small Cell Lung Cancer Receiving Immunotherapy
Source: Front Immunol. 2022 Feb 9;13:840313. doi: 10.3389/fimmu.2022.840313 (PMC8863608; doi:10.3389/fimmu.2022.840313)

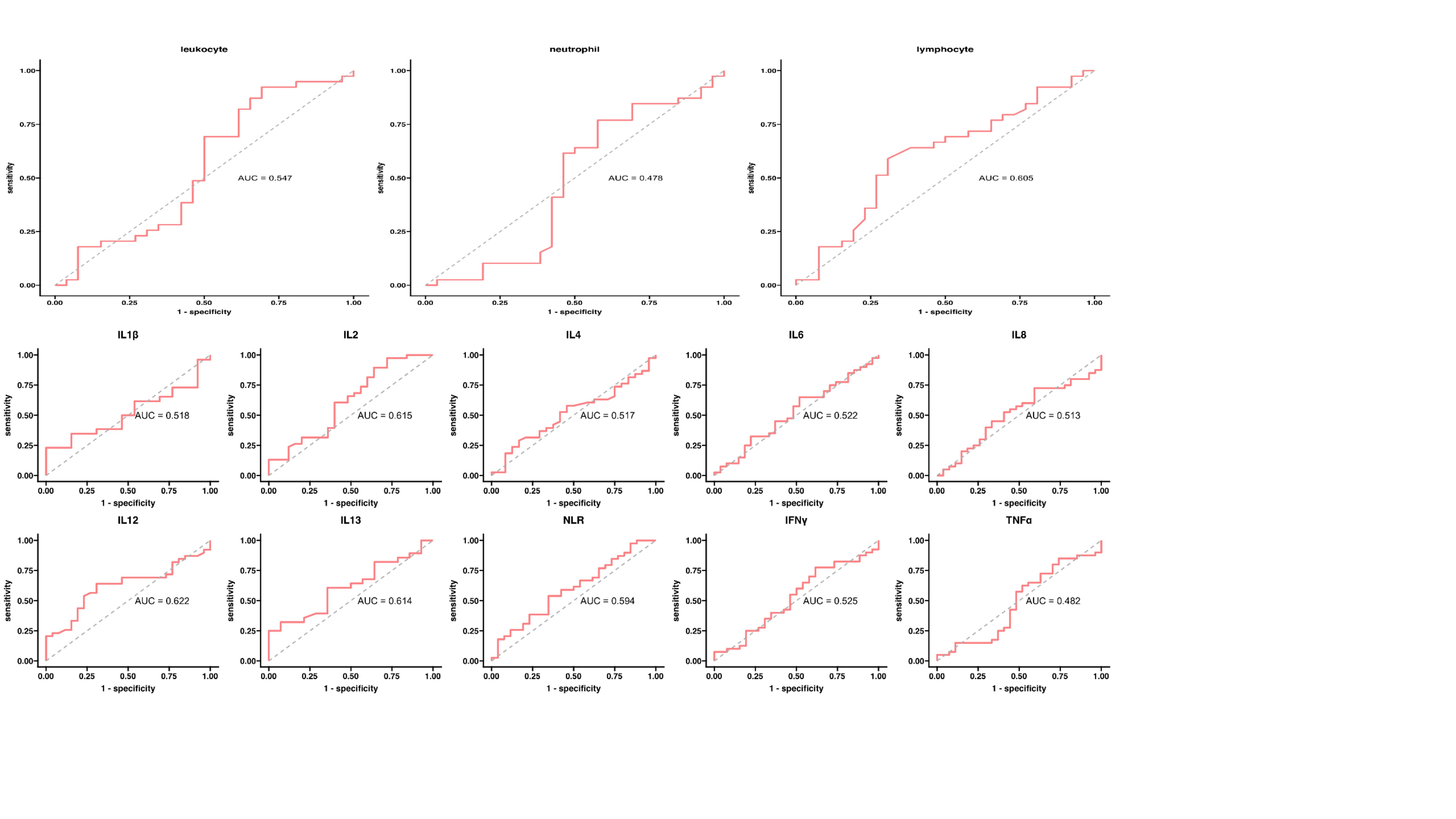

Supplement: Supplementary Figure 1 — ROC curve analysis of all blood biomarkers included and irAEs. [file Image_1.jpeg]

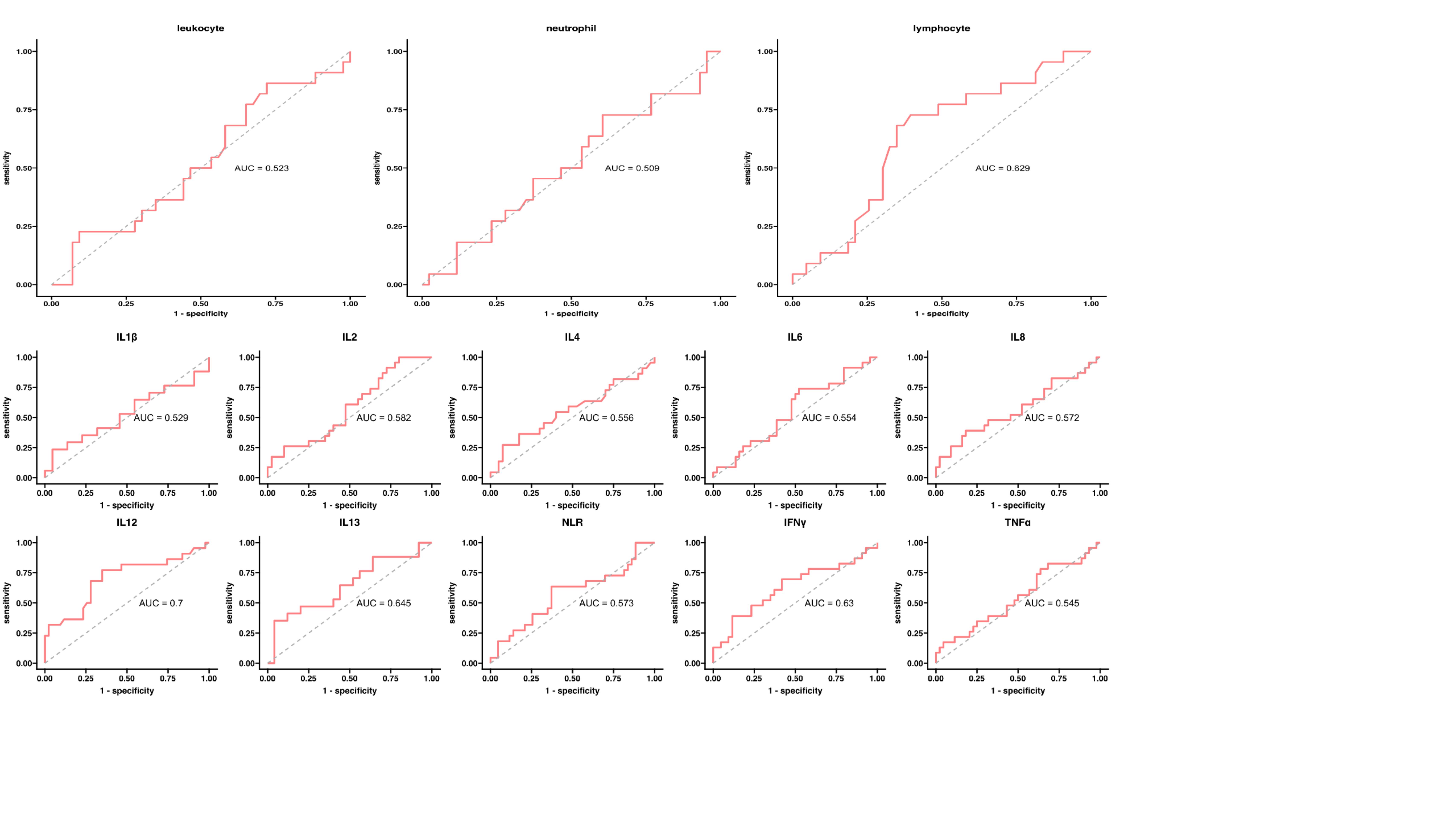

Supplement: Supplementary Figure 2 — ROC curve analysis of all blood biomarkers included and ICI-pneumonitis. [file Image_2.jpeg]

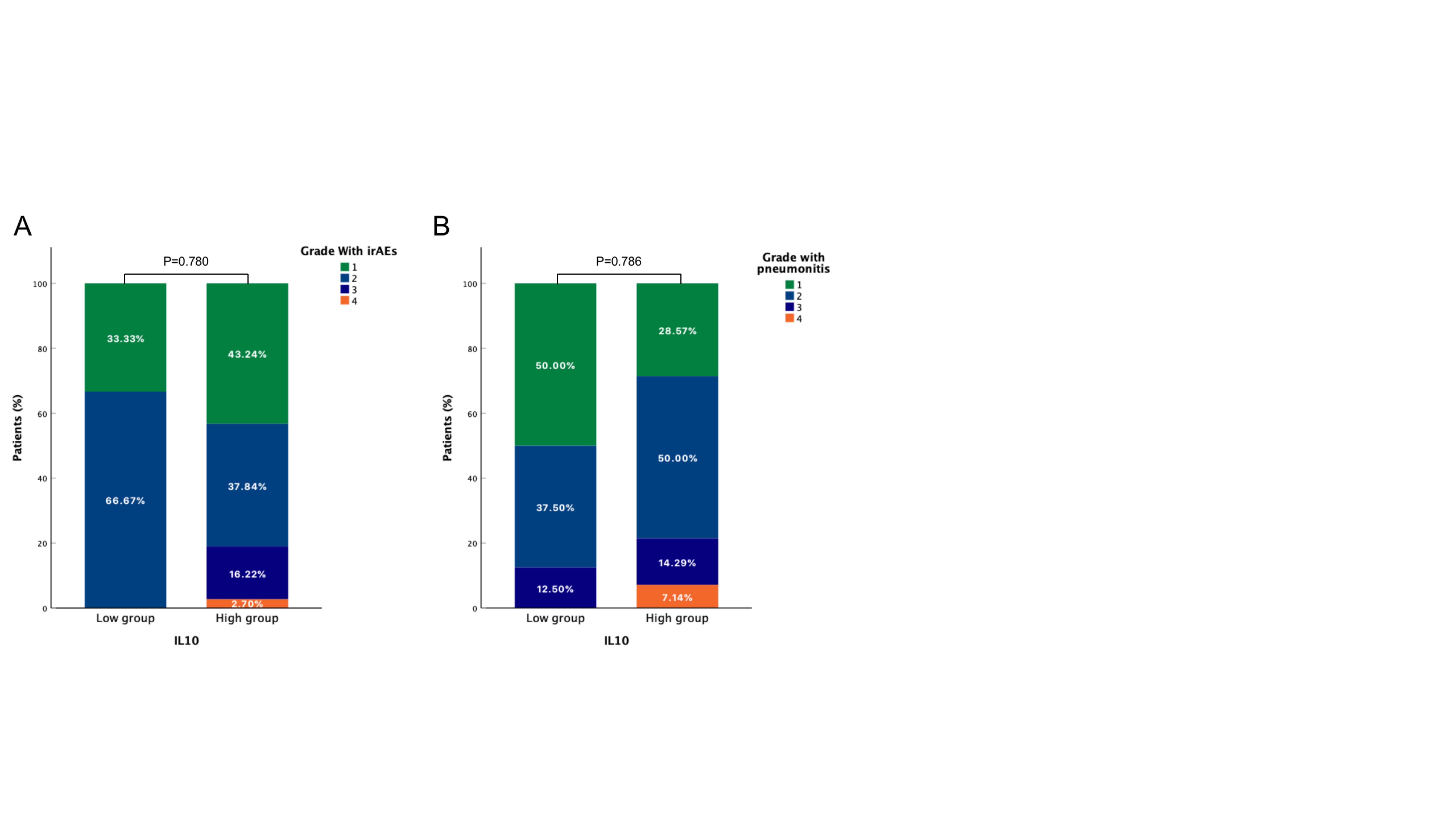

Supplement: Supplementary Figure 3 — Grade distribution of irAEs (A) and ICI-pneumonitis (B) in patients with high IL-10 and low IL-10 group. [file Image_3.jpeg]
