# Supplementary material for: Interleukin-10 Is a Promising Marker for Immune-Related Adverse Events in Patients With Non-Small Cell Lung Cancer Receiving Immunotherapy
Source: Front Immunol. 2022 Feb 9;13:840313. doi: 10.3389/fimmu.2022.840313 (PMC8863608; doi:10.3389/fimmu.2022.840313)
Supplement: Supplementary file 5 [file Table_1.docx]

| **Characteristics** | **Univariate analysis** | |  | **Multivariate analysis** | |
| --- | --- | --- | --- | --- | --- |
|  | **HR (95% CI)** | **P** |  | **HR (95% CI)** | **P** |
| Gender | 0.788 (0.346, 1.799) | 0.572 |  |  |  |
| Age(y) | 0.723 (0.386, 1.357) | 0.313 |  |  |  |
| Smoking history | 2.136 (1.135, 4.018) | 0.019 |  | 1.807 (0.943, 3.463) | 0.074 |
| Pathology | 1.613 (0.855, 3.043) | 0.140 |  |  |  |
| Stage | 0.943 (0.430, 2.068) | 0.884 |  |  |  |
| ECOG PS | 0.295 (0.069, 1.258) | 0.099 |  | 0.441 (0.095, 2.055) | 0.297 |
| PD-L1 | 1.030 (0.415, 2.558) | 0.949 |  |  |  |
| treatment lines | 1.047 (0.552, 1.987) | 0.888 |  |  |  |
| treatment strategy | 0.807 (0.428, 1.524) | 0.509 |  |  |  |
| Brain metastasis | 0.930 (0.360, 2.400) | 0.881 |  |  |  |
| Liver metastasis | 2.739 (0.958, 7.832) | 0.060 |  | 2.279 (0.739, 7.027) | 0.152 |
| Bone metastasis | 1.063 (0.552, 2.044) | 0.856 |  |  |  |
| IL-10 | 5.537 (1.689, 18.152) | 0.005 |  | 4.458 (1.329, 14.953) | 0.015 |

Supplementary Table 1. Cox proportional hazard regression model for irAEs in patients

Characteristics with P-value ≤ 0.1 in univariate models were analyzed in multivariate analysis model.

Supplementary Table 2. Chi-square test of change cytokines in patients with dynamic monitoring

| **Characteristics** | **All (%)** | **Without irAEs (%)** | **With irAEs (%)** | **P** |
| --- | --- | --- | --- | --- |
| IL-2 |  |  |  | 0.135 |
| Low group | 16 (51.6) | 8 (72.7) | 8 (40) |  |
| High group | 15 (48.4) | 3 (27.3) | 12 (60) |  |
| IL-4 |  |  |  | 1.000 |
| Low group | 15 (48.4) | 6 (50) | 9 (47.4) |  |
| High group | 16 (51.6) | 6 (50) | 10 (52.6) |  |
| IL-6 |  |  |  | 0.305 |
| Low group | 18 (50) | 9 (64.3) | 9 (40.9) |  |
| High group | 18 (50) | 5 (35.7) | 13 (59.1) |  |
| IL-8 |  |  |  | 0.733 |
| Low group | 18 (50) | 8 (57.1) | 10 (45.5) |  |
| High group | 18 (50) | 6 (42.9) | 12 (54.5) |  |
| IL-10 |  |  |  | 0.015 |
| Low group | 18 (50) | 11 (78.6) | 7 (31.8) |  |
| High group | 18 (50) | 3 (21.4) | 15 (68.2) |  |
| IL-12 |  |  |  | 1.000 |
| Low group | 17 (50) | 7 (53.8) | 10 (47.6) |  |
| High group | 17 (50) | 6 (46.2) | 11 (52.4) |  |
| IFN-γ |  |  |  | 0.086 |
| Low group | 18 (50) | 10 (71.4) | 8 (36.4) |  |
| High group | 18 (50) | 4 (28.6) | 14 (63.6) |  |
| TNF-α |  |  |  | 0.733 |
| Low group | 18 (50) | 8 (57.1) | 10 (45.5) |  |
| High group | 18 (50) | 6 (42.9) | 12 (54.5) |  |

Supplementary Table 3. Univariate and multivariate logistic regression analysis for the risk factors of irAEs in patients with dynamic monitoring

| **Characteristics** | **Univariate analysis** | |  | **Multivariate analysis** | |
| --- | --- | --- | --- | --- | --- |
|  | **OR (95% CI)** | **P** |  | **OR (95% CI)** | **P** |
| IL-2 | 4.000 (0.807, 19.818) | 0.090 |  |  |  |
| IL-4 | 1.111 (0.262, 4.719) | 0.886 |  |  |  |
| IL-6 | 2.600 (0.651, 10.384) | 0.176 |  |  |  |
| IL-8 | 1.600 (0.414, 6.177) | 0.495 |  |  |  |
| IL-10 | 7.857 (1.651, 37.403) | 0.010 |  | 5.712 (1.088, 29.993) | 0.039 |
| IL-12 | 1.283 (0.321, 5.134) | 0.724 |  |  |  |
| IFN-γ | 4.375 (1.027, 18.629) | 0.046 |  | 2.359 (0.464, 12.006) | 0.301 |
| TNF-α | 1.600 (0.414, 6.177) | 0.495 |  |  |  |

Characteristics with P-value <0.05 in univariate models were analyzed in multivariate analysis model.

Supplementary Table 4. Baseline characteristics of patients treated with ICIs in pneumonitis

| **Characteristics** | **Number of cases** | **Without pneumonities** | **With pneumonities** | **P** |
| --- | --- | --- | --- | --- |
| Gender |  |  |  | 0.287 |
| Female | 9 (13.4) | 4 (9.1) | 5 (21.7) |  |
| Male | 58 (86.6) | 40 (90.9) | 18 (78.3) |  |
| Age(y) |  |  |  | 0.612 |
| < 65 | 35 (52.2) | 22 (50) | 13 (56.5) |  |
| ≥ 65 | 32 (47.8) | 22 (50) | 10 (43.5) |  |
| Smoking history |  |  |  | 0.006 |
| Never | 44 (65.7) | 34 (77.3) | 10 (43.5) |  |
| Anytime | 23 (34.3) | 10 (22.7) | 13 (56.5) |  |
| Pathology |  |  |  | 0.169 |
| Squamous carcinoma | 34 (50.7) | 25 (56.8) | 9 (39.1) |  |
| Adenocarcinoma | 33 (49.3) | 19 (43.2) | 14 (60.9) |  |
| Stage |  |  |  | 0.368 |
| Postoperative recurrence | 16 (23.9) | 12 (27.3) | 4 (17.4) |  |
| III/IV | 51 (76.1) | 32 (72.7) | 19 (82.6) |  |
| ECOG PS |  |  |  | 1.000 |
| 0 | 2 (3) | 1 (2.3) | 1 (4.3) |  |
| ≥ 1 | 65 (97) | 43 (97.7) | 22 (95.7) |  |
| PD-L1 |  |  |  | 0.629 |
| Not detected | 17 (42.5) | 13 (44.8) | 4 (36.4) |  |
| Detected | 23 (57.5) | 16 (55.2) | 7 (63.6) |  |
| treatment lines |  |  |  | 0.234 |
| 1 | 27 (40.3) | 20 (45.5) | 7 (30.4) |  |
| ≥ 1 | 40 (59.7) | 24 (54.5) | 16 (69.6) |  |
| treatment strategy |  |  |  | 0.620 |
| Monotherapy | 38 (56.7) | 24 (54.5) | 14 (60.9) |  |
| Combination therapy | 29 (43.3) | 20 (45.5) | 9 (39.1) |  |
| Brain metastasis |  |  |  | 0.935 |
| No | 60 (89.6) | 40 (90.9) | 20 (87) |  |
| Yes | 7 (10.4) | 4 (9.1) | 3 (13) |  |
| Liver metastasis |  |  |  | 0.890 |
| No | 63 (94) | 42 (95.5) | 21 (91.3) |  |
| Yes | 4 (6) | 2 (4.5) | 2 (8.7) |  |
| Bone metastasis |  |  |  | 0.824 |
| No | 42 (62.7) | 28 (63.6) | 14 (60.9) |  |
| Yes | 25 (37.3) | 16 (36.4) | 9 (39.1) |  |
| **Blood biomarkers** Median (1st quartile, 3rd quartile) |  |  |  |  |
| Leukocyte | 6.57 (5.71, 8.46) | 6.57 (5.61, 8.46) | 6.68 (5.82, 8.51) | 0.760 |
| Neutrophil | 4.48 (3.47, 6.05) | 4.48 (3.48, 5.94) | 4.45 (3.40, 6.16) | 0.906 |
| Lymphocyte | 1.36 (1.11, 1.94) | 1.53 (1.11, 1.99) | 1.22 (1.10, 1.57) | 0.091 |
| IFN-γ | 9.15 (6.05, 15.86) | 8.33 (5.96, 12.28) | 11.57 (7.56, 20.79) | 0.084 |
| IL-10 | 1.15 (0.72, 1.92) | 1.00 (0.66, 1.64) | 1.64 (1.07, 2.31) | 0.017 |
| IL-12p70 | 0.16 (0.08, 0.27) | 0.12 (0.08, 0.21) | 0.21 (0.16, 0.51) | 0.009 |
| IL-13 | 1.44 (1.04, 2.15) | 1.44 (0.91, 1.74) | 1.53 (1.17, 4.08) | 0.115 |
| IL-1β | 0.13 (0.05, 0.29) | 0.12 (0.05, 0.26) | 0.13 (0.05, 0.50) | 0.769 |
| IL-2 | 0.26 (0.16, 0.40) | 0.25 (0.13, 0.40) | 0.26 (0.17, 0.61) | 0.284 |
| IL-4 | 0.02 (0.01, 0.03) | 0.02 (0.01, 0.03) | 0.02 (0.01, 0.04) | 0.466 |
| IL-6 | 3.66 (1.81, 6.73) | 2.95 (1.71, 6.61) | 3.78 (1.83, 7.32) | 0.468 |
| IL-8 | 5.47 (3.48, 8.86) | 5.38 (2.86, 7.10) | 5.65 (3.78, 14.08) | 0.335 |
| TNF-α | 4.93 (3.81, 6.19) | 4.86 (3.65, 6.08) | 4.96 (3.99, 6.56) | 0.544 |

Supplementary Table 5. Cox proportional hazard regression model for ICI-pneumonitis

| Characteristics | Univariate analysis | |  | Multivariate analysis | |
| --- | --- | --- | --- | --- | --- |
|  | HR (95% CI) | P |  | HR (95% CI) | P |
| Gender | 0.749 (0.275, 2.043) | 0.573 |  |  |  |
| Age(y) | 0.658 (0.287, 1.511) | 0.324 |  |  |  |
| Smoking history | 4.382 (1.792, 10.715) | 0.001 |  | 4.215 (1.570, 11.319) | 0.004 |
| Pathology | 1.461 (0.628, 3.396) | 0.379 |  |  |  |
| Stage | 1.260 (0.423, 3.747) | 0.678 |  |  |  |
| ECOG PS | 0.278 (0.036, 2.162) | 0.221 |  |  |  |
| PD-L1 | 1.140 (0.320, 4.060) | 0.839 |  |  |  |
| treatment lines | 1.332 (0.542, 3.277) | 0.532 |  |  |  |
| treatment strategy | 0.642 (0.275, 1.497) | 0.305 |  |  |  |
| Brain metastasis | 0.771 (0.226, 2.636) | 0.679 |  |  |  |
| Liver metastasis | 2.365 (0.540, 10.360) | 0.253 |  |  |  |
| Bone metastasis | 1.611 (0.654, 3.964) | 0.3 |  |  |  |
| IL-10 | 7.393 (1.717, 31.832) | 0.007 |  | 14.015 (1.794, 109.507) | 0.012 |
| IL-12 | 3.312 (1.214, 9.032) | 0.019 |  | 1.702 (0.611, 4.744) | 0.309 |

Characteristics with P-value ≤ 0.1 in univariate models were analyzed in multivariate analysis model.

Supplementary Table 6. logistic regression analysis for the risk factors of pneumonitis in patients with dynamic monitoring

| **Characteristics** | **Univariate analysis** | |
| --- | --- | --- |
|  | **OR (95% CI)** | **P** |
| IL2 | 6.12 (1.02-36.89) | 0.048 |
| IL4 | 1.25 (0.26-5.94) | 0.779 |
| IL6 | 0.74 (0.16-3.38) | 0.701 |
| IL8 | 2.50 (0.51-12.14) | 0.256 |
| IL10 | 5.09 (0.89-29.27) | 0.068 |
| IL12 | 1.94 (0.38-9.88) | 0.423 |
| TNFα | 1.35 (0.30-6.13) | 0.701 |
| IFNγ | 2.50 (0.51-12.14) | 0.256 |

Supplementary Table 7. Cox proportional hazard regression model for ICI-pneumonitis in dynamic monitoring

| **Characteristics** | **Univariate analysis** | |
| --- | --- | --- |
|  | **HR (95% CI)** | **P** |
| IL2 | 1.94 (0.38-10.01) | 0.426 |
| IL4 | 1.71 (0.40-7.25) | 0.468 |
| IL6 | 0.50 (0.12-2.10) | 0.344 |
| IL8 | 1.61 (0.38-6.76) | 0.517 |
| IL10 | 2.16 (0.43-10.79) | 0.350 |
| IL12 | 1.76 (0.33-9.37) | 0.505 |
| TNFα | 0.83 (0.21-3.34) | 0.794 |
| IFNγ | 1.01 (0.23-4.38) | 0.994 |
